# “I Have Some Sense of Loss but More Sense of Self”: A Qualitative Content Analysis of Chinese University Emerging Adults’ Personal Life Stories

**DOI:** 10.3389/fpsyg.2021.765440

**Published:** 2021-11-03

**Authors:** Hua Chen, Ying Wang, Zengmei Liu

**Affiliations:** ^1^School of International Education, Shandong University of Finance and Economics, Jinan, China; ^2^School of Foreign Languages and Literature, Shandong University, Jinan, China

**Keywords:** personal life stories, growth, social support, university emerging adults, meaningful literacy

## Abstract

The longitudinal, qualitative study aimed to explore the lived experiences of Chinese university emerging adults by analyzing their personal life stories, reflective journals, and semi-structured interview data collected over a 2-year period. A qualitative content analysis was used to identify five themes: maturity, academic performance, interpersonal communication skills, social support network, and sense of loss. The study found that the growth in emerging adulthood was dynamic, positive, and multifaceted. The study demonstrated that writing personal life stories, as a practice of meaningful literacy instruction in EFL contexts, helped students to record a memorable past, reconcile with the past, and strive for personal growth. These findings suggest that emerging adults’ personal growth be stressed in foreign language teaching to promote Chinese university emerging adults’ growth and development. It is also suggested that personal life stories be used in foreign language teaching under the guidance of meaningful literacy instruction.

## Introduction

University life is a developmental period and an important part of the path to adulthood for university students during the emerging adulthood (ages 18–25). Positive growth is crucial for university students to transform from adolescence to emerging adulthood. In the process of transition and identity formation, university students gain independence from their families (Aquilino, 2006), adapt to the university life, deal with more rigorous academic demands, build new social networks, and finally establish an identity (e.g., Arnett, 2000, 2004; Berzonsky and Kuk, 2005; McAdams and Zapata-Gietl, 2015; Vosylis et al., 2018). It is also in this process that they experience changes, encounter difficulties, and sometimes have a sense of loneliness. A large population of Chinese university students voluntarily write their own stories on web-based tools, such as blogs and WeChat. As far as the university teachers are concerned, how to help students to go through their emerging adulthood remains an important task. Accordingly, in classroom contexts, some teachers have utilized the genre of poetry as a practice of meaningful literacy instruction (Hanauer, 2012, 2015; Iida, 2016; Kim and Park, 2020), for narratives can help student narrators to “utilize memory, imagination and personal experience to explore and understand the self” (Hanauer, 2012, p. 108). Another important reason is that narratives serve the purpose of ruminating self-related actions, such as self-disclosure, self-reflection, and self-critique by thematizing and displacing themselves in the past time and space (Bamberg, 2010), while the individuality of experience offers opportunities of self-presentation, helps narrators capitalize on the potential for growth, and facilitates their personal growth (Iida, 2016).

Recent studies have examined the functions of personal life stories or the lived experiences of different groups (e.g., Tavernier and Willoughby, 2012; Hass et al., 2014; Steenbakkers et al., 2016) and demonstrated the pedagogical implications of meaningful literacy instruction (e.g., Hanauer, 2012, 2015; Iida, 2016; Kim and Park, 2020). However, lived experiences of emerging adults have been mostly researched in L1/L2 contexts, and personal life stories with emerging adults at university have been an under-researched area in Chinese EFL contexts, although it has been stressed that life stories play an important role for identity construction (e.g., Pasupathi et al., 2007; Thomsen et al., 2020). Given the role of emerging adulthood and writing personal life stories in the personal growth and development of Chinese university students, it is of importance to have a more comprehensive understanding of emerging adults in Chinese universities. It is also important to emphasize the role of personal life story writing in EFL contexts, as writing in a foreign language is difficult and even challenging for most EFL learners and need self-reflection and self-exploration in the process of writing. Therefore, with the scaffolding of EFL teachers, students can better explore themselves in a meaningful writing activity—personal life stories. Meaningful literacy instruction has recently gained increasing attention in EFL writing contexts by focusing on the genre of poetry, an investigation of the genre of personal life stories allows EFL students to have another opportunity to reflect on their lived experiences and express their thoughts in English, which thus might contribute to the personal growth and writing competence in EFL contexts.

Against this backdrop, our longitudinal study aims to explore the lived experiences of Chinese emerging adults through analyzing their personal life stories, reflective journals, and interview data. With multiple data sources, consisting of personal life stories at different periods of their life, ranging from primary school, junior/senior high schools to university, and complementing with interviews conducted in Chinese to help delve into more lived experiences of Chinese emerging adults, this study is expected to shed important light on the personal growth of university students by incorporating meaningful literacy instruction in EFL writing contexts.

## Literature Review

### Emerging Adults

Emerging adulthood (ages 18–25) is characterized by “identity explorations, instability, self-focus, feeling in-between, and possibilities” (Arnett, 2004, p. 8). Recent studies on emerging adults have primarily focused on their well-being (Adler et al., 2012; Thomsen et al., 2020), stability in narrative identity (Sengsavang et al., 2018; Thomsen et al., 2020), values (Barni et al., 2013), interpersonal relationships (Takano et al., 2011), and identity status (Crocetti et al., 2008; Hatano et al., 2016).

Studies on emerging adults’ well-being showed that personal life stories were not only more consistently related to well-being than vicarious life stories, but could lead to higher well-being with more positive emotional tone, meaning, agency, and communion (e.g., Adler et al., 2012; Thomsen et al., 2020). Studies on stability in narrative identity among emerging adults shifted the emphasis from overarching characteristics of narrative identity to the selection of events to help perceive narrators’ sense of self-continuity over time (e.g., Sengsavang et al., 2018; Thomsen et al., 2020). In terms of value transmission, Barni et al. (2013) found that emerging adults had higher similarities or transmission of values with their parents perhaps as they were more willing to accept their parents’ values (Knafo and Schwartz, 2009) and preferred a higher quality family relationship (Scabini et al., 2006). Takano et al. (2011) reported that self-rumination was associated with undergraduates’ self-perceived impaired interpersonal skills while self-reflection was related to the improved skills. According to Hatano et al. (2016), emerging adults’ identity statuses had both dark and bright sides. The study reported that emerging adults had higher scores on extroversion, consciousness, openness, and externalizing problem behaviors and indicated that they tended to establish stable identity compared with adolescents. Studies on emerging adults from the aforementioned perspectives have provided useful insights into the understanding of Chinese university students and laid a foundation for using personal life stories to delve into their personal growth in EFL contexts.

### Personal Life Stories

According to Thomsen et al. (2020, p. 2), personal life stories refer to “mentally represented narratives about individuals’ own lives, constructed to create temporarily, causally, and thematically coherent accounts of the past, present, and future” (Habermas and Bluck, 2000; McAdams, 2001), which shape who they are, how they behave (Poltera, 2010), and help identity construction and identity analysis (Bamberg, 2010; Hanauer, 2012).

Existing studies on personal life stories regarded this kind of story as a typical genre of storytelling (Raffaelli et al., 2017). One line of research has highlighted the values of writing personal life stories mostly through theoretical studies, such as helping individuals achieve a sense of identity and meaning, documenting coherence in human lives, and reflecting the awareness of autonomy (e.g., MacCurdy, 2000; McAdams and McLean, 2013; Gu, 2018). A second line of research has empirically investigated the lived experiences of adolescents and emerging adults (e.g., Tavernier and Willoughby, 2012; Hass et al., 2014). While Tavernier and Willoughby (2012) reported that turning points events could be life altering, and meaning-making and higher psychological well-being were significantly associated, McAdams and McLean (2013) found that narrative identity evolved a person’s life story. If narrators constructed stories featuring personal agency and exploration, they tended to reach higher levels of mental health, well-being, and maturity. Furthermore, Hass et al. (2014) demonstrated that the interaction among the sense of autonomy, social and instrumental support, and access to “safe havens” facilitated turning-point events. These studies have mainly focused on the functions of personal life stories or individual differences among narrators, with little attention paid to personal life stories voluntarily written by emerging adults in EFL contexts.

### Meaningful Literacy

Meaningful literacy is defined as “the approach to writing instruction that situates the appreciation and expression of the uniqueness of personal experience at the center of literacy practice” (Hanauer, 2012, p. 114). Significant within this definition is the shift from an emphasis on writing product to writers’ actual personal experience in producing the discourse.

Meaningful literacy instruction has been incorporated in classroom contexts by using the genre of poetry (e.g., Furman et al., 2007; Hanauer, 2012, 2015; Iida, 2012, 2016; Kim and Park, 2020). This pedagogy has extended both the breadth and depth of research from western countries (e.g., Hanauer, 2012, 2015) to Asian countries, such as Japan and Korea in L2 writing contexts (e.g., Iida, 2012, 2016; Kim and Kim, 2018; Kim and Park, 2020). For example, Hanauer (2012) investigated poetry in second/foreign language classrooms in a United States university and found that learners were more willing to express themselves in the process of learning a foreign language and could have the experience of self-exploration and self-reflection and produce meaningful content. Iida (2016) study pointed out that the Japanese poetry (haiku) written by L2 writers was useful in understanding their responses and emotional concerns. Furthermore, Kim and Park (2020) examined the Korean poetry (sijo) writing process of a Korean American adult and suggested that incorporating poetry writing may benefit learners in the way how they understand and express their personal experiences. This line of research suggests that meaningful literacy, as a useful pedagogical approach, could help to develop writers’ voice, emotional engagement, and ownership (Hanauer, 2015).

In brief, previous studies have mainly reported the importance of using personal life stories among different groups of people from various perspectives, compared emerging adults and adolescents, or started to use meaningful literacy in the genre of poetry to probe into student writers’ life experiences. Little has been known about what Chinese university emerging adults wrote about themselves in the genre of personal life stories in EFL contexts and how they perceived their university life. In light of the two gaps, our study aims to investigate the lived experiences through analyzing the themes of students’ personal growth in the genre of personal life stories of Chinese University students in EFL contexts. We hope that the study could add new understandings of emerging adults from a narrative perspective by analyzing personal life stories, reflective journals, and interview data in a longitudinal study. We also hope that the qualitative content analysis could help to reveal richer meanings of personal life stories written by emerging adults in Chinese EFL contexts.

## Methods

### Research Context

The present study was undertaken between the academic year of 2018 and 2020 at one university in Shandong, China. Students were required to study a compulsory course entitled Comprehensive English. The first author taught the 90-min course three times per week, in which English writing was a key element in enabling students to improve language competence and promote personal growth. Meaningful literacy instruction was used in the course as a pedagogy, and one important principle of this pedagogy is to “employ writing that utilizes memory, imagination and personal experience to explore and understand the self” (Hanauer, 2012, p. 108). This pedagogy serves the functions of providing an opportunity for students to write meaningful work and “giving a sense of depth and ownership to the writing itself” (Hanauer, 2012, p. 109). Accordingly, as teachers shoulder responsibility for fostering healthy development in students, meaningful literacy instruction was used to complement in-class teaching with an aim of having a deeper understanding of students, helping them articulate their voices, and providing effective guidance for teaching. Some students exhibited more interest in writing and voluntarily shared with the teacher and classmates their personal life stories. It was regarded as an additional opportunity for them to have more communication with the teacher and classmates and gain more from both teacher and peer feedback, thus helping them to promote their language competence and writing skills. In order to regulate this self-motivated writing activity and find more about the students, the study was conducted among a group of students voluntarily writing their personal life stories after class.

### Participants

Seven third-year full-time EFL learners (one male and six females, ages 19 to 20) voluntarily participated in the study, and written consent was obtained from the participants. Pseudonyms were used to ensure confidentiality of the participants in the study. The participants came from three different provinces, including Shandong Province (*n* = 5), Zhejiang Province (*n* = 1) and Shanxi Province (*n* = 1). All participants started learning English from Grade 3 in primary school and have learned English as a foreign language for about 11 years. They all consented to participate and were willing to write and share their personal life stories with the teacher and other participants.

### Data Sources

Data sources included two personal life stories, one reflective journal, and two interviews. Students voluntarily participated in writing two personal life stories and one reflective journal in Semester 5 after class. They reported that the average time of each piece of writing ranged between 40–50 min, and the length of their writings were within 500 English words. The personal life stories were generally written in a chronological order. The first life stories were focused more on their experiences in primary school and junior/senior high schools, and the second life stories were more on their experiences at university.

As reflection serves the function of “making sense of experience in relation to the self, others and contextual conditions and reimagining and/or planning future experience for personal and social benefit” (Ryan, 2013, p. 145), students in this study wrote reflective journals with the scaffolding of their teacher after completing writing about their personal life stories to assist in the sense making, meaning making, and future planning.

Two semi-structured interviews were conducted in Semesters 5 and 8 to get more student experiences and cover more semesters. Accordingly, all participants were invited to narrate the important points of the previously written personal stories or other personal stories they had not written because of time limit or capability of English writing, as they reported that they were unable to express themselves thoroughly in English. The first interview aimed to help understand students’ personal life experiences, which they could not thoroughly express in their writings. The second interview aimed to help gain more nuanced understanding of emerging adults’ personal growth before they graduate from the university through asking students to recall their third year and fourth year life experiences and changes. Accordingly, a pilot study was conducted and the piloting of the interview questions showed that the questions were suitable for the participants. The interviews were conducted in Chinese so that students can describe their real-thinking and were individually audio-recorded and transcribed verbatim. Students were given prompts in the process of interview, and each interview lasted appropriately 30–40 min.

### Data Collection and Analysis

Personal life stories and reflective journals were collected in Weeks 5, 10, and 15 in Semester 5, 2018, and two semi-structured interviews (*n* = 14) were conducted in Week 16, Semester 5, 2018 and Week 16, Semester 8, 2020, respectively. Students wrote more than 1 story in each writing, and 56 stories were collected. Students shared more thoughts, feelings, and emotions in their reflective journals, expressed new viewpoints, or talked about personal life stories in the Chinese-conducted interviews, which could better reveal students’ personal life experiences.

A qualitative content analysis was utilized to group categories, which went through a process of decontextualization, recontextualization, categorization, and compilation (Bengtsson, 2016). Qualitative content analysis software NVivo 12 was used to help identify the themes of personal life stories. The first two authors participated in coding, analyzing, and generalizing the themes of the personal life stories (Kappas, 0.84). If there were any discrepancies, they consulted the third author to reach a final decision. The first two authors read all data repeatedly and took coding notes to generalize, classify, and elicit meaning units from data based on the study conducted by Polit and Beck (2009). A total of 199 meaning units addressing the aim of the study were extracted from the personal life stories, reflective journals, and interview data. The meaning units consisting of phrases and sentences were condensed, and 25 categories and five themes were identified, respectively. It is hoped that the identified themes can help analyze the personal life stories in its entirety.

### Findings

The qualitative content analysis showed that students were interested in writing some common topics to describe their personal experiences and express their voices. Their growth and development at university was dynamic, positive, and multifaceted. Five themes and 25 categories are presented in Table 1.

**TABLE 1:** Overviews of themes (*n* = 5) and categories (*n* = 25) identified from the analysis of the personal life stories, reflective journals, and interviews.

Theme	Category
Maturity	Personality
	Self-positioning
	Awareness raising
	Self-autonomy
	Agency
	Decision-making
	Reflective ability
Academic performance	Goal setting
	Foreign language learning
	Content-based course learning
	Academic expertise
Interpersonal communication skills	Relationship initiation
	Relationship maintenance
	Communication abilities
Social support network	The institution
	Family members
	Teachers
	Classmates and friends
	Online resources
Sense of loss	New environment
	Family situations
	Difficulties with interpersonal relationships
	Difficulties with studies
	Dissatisfaction with self
	Loneliness

### Maturity

University students grew mature in terms of personality, self-positioning, awareness raising, self-autonomy, agency, decision-making, and reflective ability. The maturity indicated their personal growth and benefited the transition from adolescence to emerging adulthood.

The focus on personality was evident in all of the personal life stories, reflective journals, and interviews. The past experiences and new environment contributed to the positive changes in personality among university students. The entry into university and relationship initiation at university carried important implications for identity formation, construction, and development. This can be illustrated by the case of Susu. In the first round of interview, she said that she had made an active attempt to change her personality:

I was an inactive girl when I was in junior and senior high schools. I did not want to do homework and was unwilling to participate in any activities. I finished my homework until 2 a.m. in the early morning. That was bad feeling, but I could not control myself at that time. Now I sit in the first row, and I want to participate in class discussions. I have been trying to become an active student (Susu, interview).

Students expressed that a higher degree of self-positioning and self-awareness, particularly the changed attitudes and habits, facilitated the establishment of better selves, as illustrated from students’ interviews:

I did become much more mature, especially in the way of interpersonal relationships. I am different from the past. I looked at myself again and started to make new friends. I want to say that I like myself better (Linlin, interview).

In the process of writing my personal life stories, I knew myself better. In the past, I focused on facts, but now I start to think about the reasons behind the facts. I have the awareness of looking at things more comprehensively. I also knew how to communicate with others better (Yuyu, interview).

Maturity was also reflected in developing autonomy (i.e., academic autonomy and emotional autonomy), especially time management skills. Students went through a dynamic process of struggling at university. This can be illustrated by the case of Miaomiao, who identified herself as a highly independent student.

I did not know how to allocate time properly when I was a freshman. One day when I read “Time is life. When the idle man kills time, he kills himself,” I became attentive and alert to timing. As time goes by, I have improved the skills of time allocation a lot (Miaomiao, personal life story).

Students’ maturity in agency and decision-making were identified in the study. They became more independent rather than relying on their parents, teachers, or peers as in the past. Students A and D became decisive and confident in these two aspects after entering university, as shown in the personal life stories:

The new environment changed my attitudes toward learning. I think university is a start and an important part of my life. The new experience at university pushed me to reflect on my past constantly (Junjun, personal life story).

I live far away from parents now. I have more chances of making my own decisions after coming to university. My life is full of happiness, sadness, satisfaction and dissatisfaction. I often reflect on my relations with others. I have developed mature interpersonal skills. The experiences will help me become a more responsible person (Linlin, personal life story).

Reflective journals, together with personal life stories, enabled students to have opportunities to reflect on themselves. For instance, Tongtong admitted that she had acquired better reasoning power, and she can take different perspectives to understand her past and see herself more comprehensively after writing personal life stories.

Writing life stories stimulated the awareness of how I look at myself. I never thought of looking at my changes in the past. Now I have new understandings of myself in and through writing. I knew more about my weaknesses and strengths, and I have learned new coping strategies at university. This is a valuable part of my life (Tongtong, reflective journal).

### Academic Performance

The second theme was related to students’ academic performance, including goal-setting, foreign language learning, content-based course learning, and academic expertise. Students centered on the reasons for choosing their favorite major, the difficulties with and progress in learning a foreign language and content-based courses, and the comparisons of learning styles between high school and university.

Students highlighted the importance of goal-setting at university. They wrote that they must set a goal from the aspects of learning styles, interpersonal communications, and social activities. They therefore need to adjust themselves to the new challenges and changes, as demonstrated by Yuyu’s writing “After graduating from my senior high school, I always think that university life is varied and relaxing. Surprisingly, the pressure lies in every corner at university. I then set many goals” (Yuyu, personal life story).

Foreign language learning is a common concern for Chinese university students, as they regard proficiency in learning foreign languages as an indicator of academic achievement and passing language proficiency tests, such as CET-4 and CET6, as an important task at university, which may facilitate their job application or graduate program application. Junjun narrated the challenges and subsequent changes in foreign language learning, which he regarded as a meaningful process of his growth and development at university.

My first progress at university is that I improved my English a lot. At the beginning, I was not confident, and I did not want to share my stories with others. After I wrote my stories in English, I found writing about myself was very interesting and useful. I also found that sharing with others brought joy to me. It was an opportunity to share feelings and improve language proficiency. I was then willing to share my stories with others. I made progress in language learning and became a more active English language learner (Junjun, personal life story).

Content-based course learning is also a common concern for university students, as most students apply for postgraduate programs, in which they need submit the transcripts of different academic courses. Yuyu wrote in her reflective journal:

I realized that it was a kind of waste of time if I did not change myself. I wanted to further my studies. There were many courses, which were challenging for me. I started to go to the library rather than indulging myself in playing computer games in the dorm. I worked hard to get a high score. I found this was interesting and built my confidence for the future (Yuyu, reflective journal).

The concern with academic expertise was another theme of academic performance. Students realized the relationships between academic expertise and realization of dreams, as academic expertise improvement facilitated attaining the goal of becoming a postgraduate. This can be evidenced by students’ interviews:

I worked hard, acquired knowledge, and improved academic expertise at university. I believe that the mastery of academic expertise has life-long influences on me (Linlin, interview).

In the process of reading books, using online resources, and attending lectures, I realized that academic expertise has a lot to do with my future. This is beneficial for me to have a chance to successfully apply for a postgraduate program (Tongtong, interview).

### Interpersonal Communication Skills

Students regarded interpersonal communication as an important aspect of positive growth accompanied by challenges and barriers. The categories included relationship initiation, relationship maintenance, particularly repairing an important relationship, and communication skills. Students’ self-rumination helped them overcome interpersonal communication problems and enhanced personal growth through getting involved in joining student organizations and social activities.

Relationship initiation, particularly making new friends to participate in social activities or seek support, exerted strong influences on their university life. Most students were successfully involved in university activities, which extended their friendship networks and deepened the understanding of friendship. For example, “I have made great progress in interacting with both classmates and friends. When I had difficulty, I think about the reasons why I had the difficulty. If I cannot solve it by myself, I would talk with my friends. I find that talking to friends is helpful” (Linlin, personal life story).

Relationship maintenance is important for student growth as in this process of learning how to maintain good relationships, they learn to cope with negative experiences and emotions with the assistance of friends. This kind of interpersonal communication is important for university students too. For example, “I have improved the interpersonal communication skills. If I do not agree with other people, I can look at things from their perspectives” (Lili, interview). As university students grow, they grasp more skills of maintaining suitable relationship with their family members. Linlin, who identified herself as a student reluctant to communicate with her mother, gradually regained her willingness to interact with her mother in the process of maintaining interpersonal relationships, as demonstrated by the interview data:

I did not talk with my mother very often when I was a high school student. After entering university, I had some difficulties with interpersonal relationships. I realized I should first have a good relation with my mother. When I had no difficulty talking with my mother, I improved my interpersonal relations with my classmates (Linlin, interview).

The interview data demonstrated the strong positive effect of friends on the family bond repair for university students. Linlin highlighted the importance of interpersonal relations and described similar events in personal life stories, reflective journals, and interviews.

### Social Support Network

Social support network included support from the institution, family members, teachers, classmates and friends, and online resources. Social support provided emotional and instrumental support, such as relieving stress, offering encouragements, building confidence, and accessing information. For instance, Junjun wrote that “Compared with my high school classmates, I am a lucky one. University is a place where I could have a new life and environment” (Junjun, reflective journal). Lili said that “University is important for me, and the experiences at university are valuable in my life” (Lili, interview). The data indicated that students realized the important role of university life.

Family support was a protective factor for adjustment and pursuit of life after the entry into university for most university students. Support was given to students in different forms and yielded different responses. Most students realized the positive role of family support. For example, “My parents always encouraged me to keep confident and take a future-oriented perspective” (Junjun, personal life story). However, the student whose parents did not have much time and communication with her said “My father seldom talked to me. He said he did not know much about my studies and cannot give me any suggestions. At that time, I was sad, and I would ask my teacher and friends for help” (Miaomiao, interview).

Support from teachers facilitated the successful transition from adolescence to emerging adulthood academically, psychologically, and behaviorally.

When I was a middle school student, I did not have much confidence, and I did not have a clear goal for my future. But at that time, I worked very hard because I wanted to go to university and change my fate. I took the college English course when I was a freshman. My English teacher encouraged me to set a goal, find my interest, and express my thoughts through writing. I would ask her for suggestions when I had some questions (Junjun, personal life story).

Support from peers provided care, assistance, trust, and emotional support to the counterparts, as demonstrated by “The friends around me made me think about myself constantly. When I had difficulties, they encouraged me and helped to solve problems. I really cherish friendships” (Yuyu, reflective journal).

Online information resources played a significant role for university students, both academically and emotionally. It provided relevant online resources to help complete their academic assignments. Students also sought help or wrote blog diaries online to express their views, seek psychological help, or keep a record of their experiences, as demonstrated by Junjun:

When I have a bad mood, I like writing about my experiences and feelings on my favorite online platform. It is a way for me to cheer up. And it is an outlet for my feelings and emotions (Junjun, interview).

### Sense of Loss

In the previous themes, growth out of maturity, academic performance, interpersonal communication skills, and social support network was mentioned. The sense of loss ran through the aforementioned aspects of growth for university students, which was mainly caused by the environment changes, family situations, difficulties with interpersonal relationships and studies, dissatisfaction with themselves, and loneliness.

As a challenging life experience for students, the new environment brought a series of loss in the process of transformation, identity formation, and adaptation to university life. In high schools, they paid more attention to test scores and tried their best to go to an ideal university. They reported that communicative competence was prioritized at university, and they spend a lot of time on improving this competence. The social support they received reduced their worries about university life and themselves. For instance, Junjun expressed his sense of loss in the two rounds of interviews.

I suffered not one loss but many losses when I was a freshman. I come from a small village. It is my first time to come to a big city. It took me many hours to take a train, caught the university shuttle bus, and at last arrived at university campus. Everything was new to me, and I was not sure whether I could adjust to the new university settings (Junjun, interview 1).

In the second round of interview, the student had accepted what university meant to him.

It took me almost 3 months to get used to the university environment. It was a hard time for me, as I had to adapt to different changes, such as learning styles and living with other seven students in the same dorm. After spending 4 years here, I have got used to it, and I have made myself a new person and a better self (Junjun, interview 2).

Family situations, such as parents’ divorce or separation, had different influences on students. They changed their attitudes from viewing the events negatively during adolescence to a positive attitude toward the past experiences at university. Some of them even regarded the events as functional experiences, which manifests students’ growth, as evidenced by the identity renewal.

My parents divorced when I was eight. I lived with my father and had hardly seen my mother since then I believe that all these experiences made my life different and forced me to grow up. I know the differences between my peers and me, and I know what I should do in the future (Miaomiao, personal life story).

Difficulties with interpersonal relationships and studies were highlighted in the study, as interpersonal communication and academic performance were two important parts of university life. Students promoted personal growth in the process of experiencing the difficulties posed by communicating with others and acquiring knowledge at university. It was also in this process that students were dissatisfied with themselves and perceived a sense of loss. For instance, “People feel disappointed if there is a big gap between hope and reality. I also felt disappointed with myself when I did not get a good score or was criticized by the peers” (Miaomiao, personal life story).

However, sometimes students’ sense of loss promoted their personal growth. They realized the values of life experiences and writing about their sense of loss somewhat promoted their personal growth, after which they were more willing to expose themselves through writing in English. They made the decision that they were ready to overcome difficulties, accept the reality, and strive for a better self. “After I came to university, I gradually realized my family hardships, and I had some pressure. I started to work part-time. I worked hard, spent less money, and became more confident” (Lili, interview).

Students’ sense of loss could be alleviated by writing personal life stories, as demonstrated by the interview data. Students reported that they regarded writing their personal life stories as an opportunity to express themselves, reconcile with the past, and find their own weaknesses and strengths. They emphasized in the interviews that they gradually started to show their inner selves to other people and found the process engaging. For example, “Writing personal life stories is interesting. I spent some time choosing some appropriate events. This process helped me reflect on my past. It is a valuable experience” (Junjun, interview) and “I know myself better after writing my personal life stories. Writing stories helps me set up new goals, control my temper, and improve interpersonal communication skills” (Tongtong, interview).

Another student mentioned the importance of writing personal life stories after class.

Before writing, I thought a lot about what to write. Many old memories came to me. I wrote my stories down. I sank into deep thoughts. I think writing personal life stories is very meaningful. I did not mind spending time thinking about what to write and how to write, because I have an opportunity to think over my past (Susu, interview).

The growth at university motivated students to pursue dreams, take more responsibilities, boost confidence, take further actions in writing their inner voices, and finally gain a sense of self. For instance, “The development at university motivated me to make more progress” (Yuyu, personal life story). Students have developed an awareness of self in describing their growth, such as “The most important growth at university was the sense of self. I know who I was, what kind of person I want to become, and what plans I need to make for my future” (Tongtong, interview), and “I know myself better. I thought over what happened in the past. This reminded me of thinking about myself again and again. I also tried to do better in my studies” (Lili, interview).

## Discussion

A qualitative content analysis of the personal life stories, reflective journals, and interviews was used to give voice to students’ views of their university life. The study gave evidence to the use of meaningful literacy in helping writers to explore themselves and develop voice in EFL contexts, which was similar as previous studies (Hanauer, 2012, 2015). This study identified five themes, i.e., maturity, academic performance, interpersonal communication skills, social support network, and sense of loss. All the themes demonstrated the positive, dynamic, and multifaceted growth of emerging adults at university.

The first recurring theme was students’ different aspects of maturity. Students gradually established a positive identity in the dynamic process of becoming mature. University experiences, together with writing personal life stories, enabled students to have more opportunities of self-reflection and had positive influences on their maturity. The study expands on previous studies of personal life stories (e.g., Tavernier and Willoughby, 2012; McAdams and McLean, 2013; Hass et al., 2014; Thomsen et al., 2020), which have theoretically focused on the functions or empirically examined the experiences of narrators in L1 contexts. Our study used personal life stories, together with reflective journals, and interviews to have a more comprehensive understanding of the lived experiences of Chinese university students in EFL contexts. We also found that students changed their attitudes toward academic performance, interpersonal communication skills, and social support network, which led to maturity at university.

A second recurring theme was related to academic performance. Students, especially Asian students, generally perceived academic performance as an important criterion of academic records and a way of changing their fates (Fuligni, 2001; Baumann and Hamin, 2011) and worked hard to obtain a high English score to “demonstrate their English proficiency to future employers” (Han and Hyland, 2015, p. 33). While Fuligni (2001) reported that Asian students had “stronger faith in the importance and utility of education for their adult lives” (p. 67), Baumann and Hamin (2011) argued that cultural value was one of the key drivers of academic performance. In our study, the immense changes in teaching styles and subsequent learning styles led students to adapt to the new changes and challenges. They developed confidence and perceived university life as an important stage for the realization of dreams. This notion seemed to be influenced by family situations and personal pursuit for future dreams. The hardships they experienced in the past during the adolescence had positive influences on their improvement in academic performance and helped them become a much better student. This can be explained by the fact that disclosing an emotionally intense experience was helpful (Zech and Rime, 2005).

A third recurring theme was the interpersonal communication skills. Before they were admitted to the universities, the students had had to take part in the National College Entrance Examination among a large population of adolescents. In high schools they were devoted to study and did not have much time to communicate with other people. In the process of transition from adolescence to emerging adulthood, they were not very skilled at interpersonal communication but were encouraged to participate in activities at university. The shifts brought challenges, difficulties, and sense of loss, but self-rumination helped them succeed in improving interpersonal communication skills. The findings corresponded with those of previous studies (e.g., Lyubomirsky and Nolen-Hoeksema, 1995; Takano et al., 2011). Lyubomirsky and Nolen-Hoeksema (1995) found that rumination was associated with impaired interpersonal problem solving while Takano et al. (2011) reported that self-rumination was associated with relationship-initiation skills. In our study, writing personal life stories, as a way of meaningful literacy, was itself a process of self-reflection and helped university students reflect on and repair the problems in interpersonal relationships.

A fourth important theme was the source of different social support, which mitigated the stress in interpersonal relationships and school work, relieved the emotional exhaustion, and alleviated loneliness. The source of social support is consistent with results of previous studies showing that social support contributes to emerging adults’ quality of life, such as academic achievement and emotional exhaustion within the university contexts (e.g., Li et al., 2018), teacher and peer support reducing loneliness (e.g., Chai et al., 2019), and family with affectionate parent-child relation offering warmth to help better cope with loneliness (Yang et al., 2020). We also found that social support from friends and classmates not only had direct positive effects on interpersonal relationships with peers, but also had indirect positive influences on the relations with parents. Support from classmates enabled them to have more confidence in the learning of foreign languages and content-based courses. One explanation for this phenomenon is that the participants in this study all lived away from their home and spent most of the time with friends and classmates at university. They learned how to understand other students’ perspectives and could better assess situations or feelings. Students without strong family bond may find support from teachers, friends, and classmates helpful to solve different problems. Our findings indicated that in classroom contexts, students should be provided with more opportunities to cooperate with peers and teachers, which will bring more chances of improving their university life.

The fifth theme was the sense of loss among university students. Students highlighted the sense of loss at university but expressed increased strength. They realized the powerful healing effect of writing personal life stories and admitted that university brought challenges and difficulties, including the adaptation to university life, difficulties in learning a foreign language, content-based courses, and interpersonal relationships and studies, dissatisfaction with themselves, and even loneliness. Calhoun and Tedeschi (2001) explained that there existed personal growth in the wake of losses of different kinds. Our study found that university students started to increase their strength when confronting losses in writing their experiences.

Students further emphasized the importance and usefulness of writing personal life stories. Personal life stories, as an important genre of meaningful literacy instruction, could allow a full understanding of university students as language learners, who are individuals “with a rich, extended history of personal experience” (Hanauer, 2012, p. 108). In the process of choosing appropriate events to construct identity, they utilized memory and reflected on their personal experiences to explore the self, as demonstrated by Hanauer (2012). In this sense, writing helped to record the past experiences and reconcile with the past, which was consistent with the findings of Gu (2018). Our study found that writing personal life stories led to self-reflection and self-control, and the dynamic improvement helped students get mature, set up new life goals, and become better selves. All these experiences helped to make university life more meaningful and promoted student personal growth.

## Conclusion

The study has qualitatively explored the lived experiences of university students through analyzing the written personal life stories, reflective journals, and interview data, which contributes to the literature on emerging adults’ personal growth in several ways. Contributions include identifying the common concerns of Chinese university students, which may facilitate a nuanced understanding of the growth and development of emerging adults. The study can also help teachers understand university students through their personal life stories. It is therefore suggested that EFL teachers may focus more on the writing content through incorporating personal life stories in the course curriculum. Furthermore, emerging adults’ growth was positive, dynamic, and multifaceted, indicating that researchers can build on the findings to further investigate other aspects of student personal growth and/or other stages of university life. Future research may also investigate how other genres may contribute to emerging students’ well-being and quality of life.

Notwithstanding these contributions, there are two main limitations. The first limitation is the analysis of only the themes of personal life stories. Future research thus can expand the research perspectives by incorporating individual differences, family background, gender differences, and age differences. A second limitation is that the data were collected from students majoring in social sciences and humanities. Future research can extend to students in other disciplinary fields and compare the role of personal life stories in different fields.

## Data Availability Statement

The raw data supporting the conclusions of this article will be made available by the authors, without undue reservation.

## Ethics Statement

The studies involving human participants were reviewed and approved by the Research Ethics Board of Shandong University (Reference No. ECSBMSSDU2018-1-049). The patients/participants provided their written informed consent to participate in this study.

## Author Contributions

HC analyzed the initial data and wrote the methods, findings, and discussion sections. YW and HC designed the study. YW and HC worked with ZL in further data analysis, drafting the manuscript, and revising the manuscript. All authors contributed to the article and approved the submitted version.

## Conflict of Interest

The authors declare that the research was conducted in the absence of any commercial or financial relationships that could be construed as a potential conflict of interest.

## Publisher’s Note

All claims expressed in this article are solely those of the authors and do not necessarily represent those of their affiliated organizations, or those of the publisher, the editors and the reviewers. Any product that may be evaluated in this article, or claim that may be made by its manufacturer, is not guaranteed or endorsed by the publisher.
